# City footprints and SDGs provide untapped potential for assessing city sustainability

**DOI:** 10.1038/s41467-021-23968-2

**Published:** 2021-06-18

**Authors:** Thomas Wiedmann, Cameron Allen

**Affiliations:** 1 Sustainability Assessment Program (SAP), School of Civil and Environmental Engineering, UNSW, Sydney, NSW Australia; 2 Monash Sustainable Development Institute, Monash University, Melbourne, VIC Australia

**Keywords:** Environmental impact, Sustainability, Society

## Abstract

Cities are recognised as central to determining the sustainability of human development. However, assessment concepts that are able to ascertain whether or not a city is sustainable are only just emerging. Here we review literature since the Sustainable Development Goals (SDGs) were agreed in 2015 and identify three strands of scientific inquiry and practice in assessing city sustainability. We find that further integration is needed. SDG monitoring and assessment of cities should take advantage of both consumption-based (footprint) accounting and benchmarking against planetary boundaries and social thresholds in order to achieve greater relevance for designing sustainable cities and urban lifestyles.

## Abstract (web summary)

Whether or not a city achieves absolute sustainability is difficult to assess with existing frameworks. Here the authors, in a review, show that a further integration of consumption-based accounting and benchmarking is necessary to aid the monitoring and assessment of Sustainable Development Goals in cities.

## Introduction

The UN 2030 Agenda and Sustainable Development Goals (SDGs)^1^ specify national ownership, with monitoring and implementation primarily targeted at the global and national levels. However, there is emerging practice in downscaling the SDGs to the sub-national level^2^ and for cities^3–5^. Cities in particular have been regarded as central to driving the sustainable development agenda in several ways. Cities exert a profound and far-reaching influence on the environment and societies outside their boundaries because of their population density, economic significance, affluence and ensuing global resource requirements. But cities are also centres of knowledge, technology and innovation, which makes them crucial players in any sustainability transition^6^. Key to the success of cities in implementing the SDGs will be meaningful monitoring and target setting, based on metrics that show progress towards sustainability in relative and absolute terms. However challenges remain in downscaling targets and indicators to the city level to support planning and policy in a local context^7^.

In addition to the emerging SDG city assessment literature, many studies have calculated the environmental footprints of cities to quantify their extended global impact from a consumption perspective^8–11^. This is particularly important for cities, since their populations cannot be sustained without inputs of resources and services from regional and global hinterlands^6^. Footprints are metrics that account for indirect impacts of an activity or entity. The carbon footprint of a city, for example, represents not only greenhouse gas emissions from within city boundaries but also those from production activities in regions and countries outside the city that are linked to city activities. Different scope definitions and calculation methods exist^8,12^, but typically footprints refer to impacts related to the final consumption of goods and services in one place and are therefore said to constitute consumption-based accounts (CBA)^9,13^. Footprint indicators have been described for many environmental and several social indicators^14^ and have also recently been linked to the concept of a ‘safe and just’ development space^15^. This framework combines the planetary boundaries (PB) concept, which maps out long-term sustainability thresholds for human-induced resource use and pollution based on Earth science principles^16,17^, with social and economic foundations that align with the goals and targets of the SDGs^18,19^. There is a substantial overlap between PBs and environmental SDGs^20^. Defining what is environmentally safe and socially just, helps to operationalise the SDGs based on quantifiable benchmarks. Currently, no country in the world operates fully within this safe and just operating space (SJS)^18,19^.

**Fig. 1:**
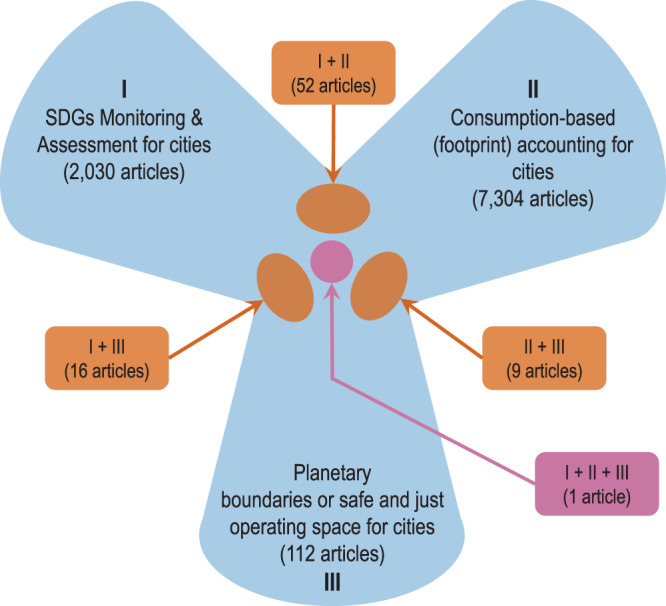
Conceptual map of the literature landscape related to this review. A search for scientific articles matching city-relevant SDG research (I), city footprint studies (II) and research on planetary boundaries or safe and just operating space related to cites (III) was conducted. Numbers of articles refer to search results in the Web of Science from 1990 to 2020. For details on search terms see the Supplementary Data (SD).

The aim of this review is to synthesise current approaches to methods, indicators and data that aim to evaluate sustainability at the city-level. Given the importance of considering trans-boundary impacts and sustainability thresholds, the focus of this review is on consumption-based (footprint) studies and their links to the SJS framework in the context of monitoring the goals, targets and indicators of the SDGs. We conceptualise the literature landscape in a framework that brings together the relevant strands of research (Fig. 1). Of particular interest for this review are studies that fall in the overlapping areas, bringing together concepts of SDGs, footprints and PBs/SJS in the context of city sustainability assessment. As will be shown, these areas exhibit major gaps in our knowledge and practice of city sustainability.

## SDGs and city sustainability assessment

The SDGs provide a comprehensive and integrated framework of 17 goals, 169 targets and 231 unique indicators designed to guide progress on sustainable development to 2030^1,21^. Assessments of progress on the SDGs have been published at various scales, including global^22^, regional^23^, national^24^, subnational^2^ and cities^4,5^. Recent reviews of the approaches and methods applied^25^ highlight that the results from such assessments will vary considerably depending upon the indictors, targets and methods applied. Science can provide assistance by defining indicator frameworks, quantifying indicators, and developing robust metrics and methods for evaluating progress^26^.

Meeting the basic needs of escalating urban populations while ensuring the integrity of critical ecosystems is one of the major challenges of our time^7^. Increasingly, cities are being conceptualised less as problems and more as drivers of sustainable development and global environmental change^7^. This important role is manifested in Goal 11 (Sustainable Cities and Communities), with its own 10 targets and 14 indicators. Many other SDGs are also relevant for sustainable cities^7^, including those relating to water (SDG 6), energy (SDG 7), sustainable consumption and production (SDG 12) and climate action (SDG 13). Giles-Corti et al.^27^ found that around 50% of the official SDG indicators could be spatially disaggregated to measure inequities within cities.

Although the SDGs are global in nature, there is increasing awareness that they have an important local dimension as they touch on many aspects administered by local governments including education, water and sanitation, waste management, health, transport etc. Achievement of SDGs will require action from cities, and examples of local councils implementing the SDGs are increasing in number, including through city SDG action plans which use the goals as a guiding framework to orient city development^28^. In these initiatives, advocacy or raising awareness is seen as an important first step to localising the SDGs. Other important elements include aligning national and local leadership, agreement on a common vision, and local access to financial resources^29^. Guides on localising the SDGs in cities are also available^30^, with recent efforts by cities documented in numerous Voluntary Local Reviews submitted to the UN, most recently for cities such as Bonn^31^, Buenos Aires^32^, Guangzhou^33^, and Sao Paulo^34^, amongst others. The SDGs have an advantage compared to many other sustainability assessment indicators and frameworks in that they emerge from a legitimate consensus of member states to the UN^7^. However, a number of theoretical and practical considerations remain regarding indicator selection, target setting and implementation^35^.

### Sustainability assessment of cities

Frameworks for assessing city sustainability pre-date the SDGs and generally follow common sustainability principles, but the wide range and complexity of the concept means that there has been no common framing. Existing assessment frameworks have been criticised for lacking integration and implementation of coherent and city-specific values, principles, goals and solutions^36^. These include not only infrastructure, housing, mobility, technology, etc. but also social well-being in specific urban contexts, recognising that social and environmental sustainability are strongly connected^6^. There has been a proliferation of indicator sets^37^, but the selection of comprehensive and relevant indicators remains challenging^36^. Monitoring methods focused on the sustainability of cities, are reviewed elsewhere^36,38,39^.

A key criticism of previous city sustainability assessments is that many use simple additive indicator models which give an overall relative city performance score, but fail to consider the role cities play in instigating global impacts of consumption and production. This has broad implications for achieving the SDGs^3,7,37,40^. While such dominant approaches can introduce competition over relative position and provide incentives to improve status, it is also important to consider absolute sustainability^41^. This is because the dominant economic growth imperative—often still viewed as being beneficial for sustainability—has in fact been shown to be incompatible with the environmental constraints of a finite planet^42–44^. Progress has recently been made to operationalise thresholds, tipping points and limits in both environmental^45^ and social systems^18,46^, but implementation in the urban context remains scant^6^.

Crises such as climate change or loss of biodiversity are often presented as global challenges, however the vast majority of actions leading to these impacts originate in cities^47^. As such, achievement of the SDGs in line with PBs will mostly be determined by actions taken in cities, and city-level assessments should provide the evidence-base for such actions. While the PBs cannot be directly applied at a local level, proper attribution of impacts from cities needs to be quantified^47^ which requires locally relevant, applied and quantitative methodologies to monitor local and global progress toward sustainability.

The interconnected impact of cities on global processes has been studied for several decades^15^. Lützkendorf and Balouktsi^35^ highlight that both production-based and consumption-based footprint approaches are needed to assess city sustainability, particularly for developed countries with very high consumption city lifestyles. Environmental footprint indicators provide a means to measure natural resource use and emissions that can also be reconciled with PBs to measure the extent to which Earth system processes are being disturbed by human activities^15^.

### Recent practice in assessing the SDGs at the city scale

The emerging literature highlights recent practice in applying the SDGs at the city-scale to assess progress, including applied case studies in the academic literature^48,49^ as well as the grey literature^4,5,50,51^. Several academic articles also address methods and challenges associated with measuring SDG progress in cities^3,27,37,48^ as well as, more specifically, for SDG 11 indicators^7^.

This literature highlights some key advantages and challenges associated with using the SDGs as a framework for evaluating progress of cities. Advantages include the integrated framing and comprehensive scope which include often neglected issues such as gender and income equality, as well as the goal-oriented approach^7,37^. City-level SDG assessments are expected to enable users such as local governments and administrations to understand their strengths and weaknesses in comparison with other cities in the world^3^.

However, identified challenges and limitations include the large number of SDG indicators, generic characteristics and limited policy relevance at the city level^37^, and the lack of standardised, open and reliable data and city-scale data collection capabilities^7,37^. Meaningful downscaling and adaptation of global or national targets and indicators to the city level to support planning and policy is also challenging^3,7^, and the SDG targets tend to assess outcomes rather than integrated policies and interventions^27^.

Another important challenge relates to the impacts of cities beyond their borders and how these should be addressed by the SDGs^37^. Recent city-scale SDG assessments often include per-capita greenhouse gas emissions metrics, however, it is unclear whether these are production- or consumption-based. Other footprint metrics are absent from these assessments and the focus is on relative performance rather than absolute sustainability, i.e. there is no consideration of global environmental limits such as the global PBs^16^.

This limitation is highlighted by Akuraju et al.^48^, who find that city-related SDG targets aim at various aspects of city sustainability which are not necessarily beneficial for global sustainability and likely conflicting with PBs^52^. There is generally a focus on techno-efficiency (such as energy reduction and energy efficiency of building stocks or transport), while neglecting the need for behavioural change in city lifestyles^40^. This echoes a concern in the broader SDG literature regarding underlying tensions between socio-economic and environmental targets in the SDGs which undermine their achievability^42,53^.

## City footprints and opportunities to monitor the SDGs

### Advances in city footprint studies

**Fig. 2:**
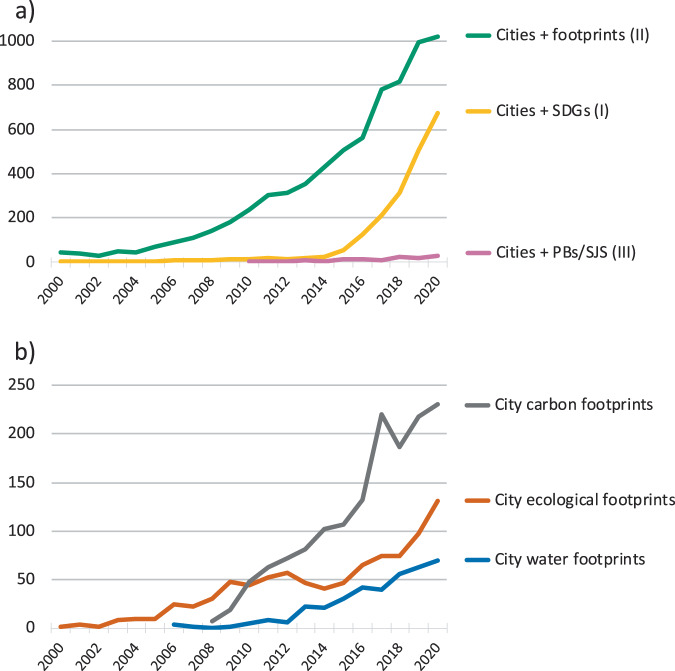
Number of published academic papers relevant to this review. The graphs show the number of published articles per year between 2000 and 2020 on topics related to cities, footprints, Sustainable Development Goals (SDGs) and planetary boundaries or safe and just operating space (PBs/SJS) as documented in the Web of Science database (for search strings see Supplementary Data). **a** Provides details of aggregate numbers in Fig. 1 on the three main literature areas of this review. **b** Provides details of aggregate numbers in Fig. 3 on selected footprint studies of cities.

Cities were a main focus of footprint studies^54^ from the beginning. The metaphor of the Ecological Footprint (EF) was first introduced in the 1990s^55^, showing clearly how much resources cities consume beyond their actual territorial boundaries^56,57^. In the late 2000s, the focus of both academia and practice began to shift towards investigating greenhouse gas emissions, with city carbon footprint (CF) studies surpassing city EF studies since 2010 (Fig. 2). This coincides with an increasing number of cities setting net-zero emissions targets and using standard protocols^58^ to comprehensively estimate all emissions from city-related activities^8,9,59^. A steady, moderate rise in water footprint (WF) studies of cities since 2010 can also be observed, whereas other footprint studies for cities remain scarce (Fig. 2 and SD).

One key message of CF studies of cities has been the extent of transboundary GHG emissions linkages. Emissions embodied in the production and supply chains of goods and services (called ‘Scope 3’ emissions^8,35,58^) are often the largest part of a city’s full GHG inventory and often found outside its physical boundaries^59,60^. For example, two-thirds of the consumption-based GHG emissions of 79 C40 cities (2.2 of 3.5 Gt CO_2_e) are imported from regions outside the cities^61^. Various carbon footprint metrics are available^9^ to quantify this carbon leakage, which is outside the legal reach of city jurisdictions^62^. Cities are more open than countries to exchanging material and financial flows with other regions, enabling their growth via global supply chains^12^. International imports are particularly important for the embodied emissions of both goods and services^63^, as well as key urban infrastructure^12^.

Another regular key finding is that cities tend to have higher shares in per-capita Scope 3 emissions than rural counterparts, because income and spending is generally higher in cities, which increases indirect, service-related emissions in particular. In contrast, the share of direct (Scope 1) emissions from household energy use and personal transport tends to be lower in dense inner-city areas^64^. A post-carbon scenario for ten European cities, developed by local city stakeholders, projects a decrease in production-based emissions (Scope 1 and 2) in all cities whereas consumption-based embodied (Scope 3) emissions are shown to increase in eight cities, due to rising affluence^65^.

Several studies investigate the WF of food and other consumption in cities, revealing the impact of industrialised^66,67^ and global^10^ supply chains on the ‘true’ water consumption of cities and the associated depletion of global water resources^67^. Depending on the exact method to calculate city WFs^11^, it is possible to trace and assess the water requirements of agriculture in one region globally with specific diets in a particular city^10,68^. While direct urban water use needs to be evaluated against local and regional water resources, it is essential to complement this with consumption-based water accounting at global scale to recognise and manage trade-offs^69^. This extends the reach and efficacy of local decision-making and can accelerate the transition to more sustainable agriculture, food systems and diets^15,66^.

### City footprints and linkages to the SDGs

**Fig. 3:**
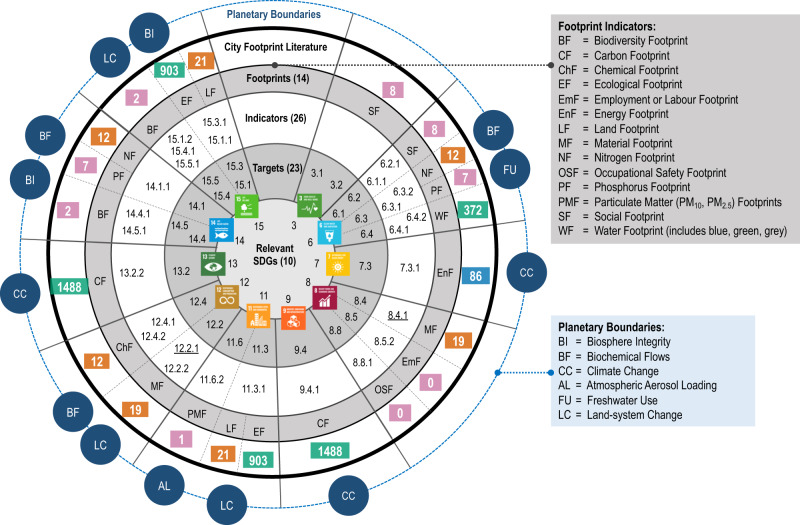
Relationship between SDGs, footprint indicators, city footprint studies and planetary boundaries. Shown are the 10 SDGs (centre), 23 SDG targets (first ring) and 26 SDG indicators (second ring) that relate to 14 specific footprint indicators (third ring), as well as the number of peer-reviewed journal articles identified relating to city footprints based on the set of query terms used in the WoS database (Methods) (fourth ring). The article numbers are colour coded (green = >100 articles; blue = 50–100 articles; orange = 10–50 articles; purple = <10 articles. Partly based on Table 3 in ref. ^15^. Note that the only official SDG indicators that include a footprint metric (MF) are 8.4.1 and 12.2.1 (underlined in the figure). The final outer ring lists the planetary boundaries that correspond to the SDGs.

The unique life-cycle and supply chain perspective of consumption-based accounting has been recognised as the main reason why footprint indicators will be essential for a consistent implementation of the SDGs^70^ and for identifying potential demand-side measures for mitigation^15,71^. Despite the great potential, the official indicator framework for the SDGs has limited coverage of footprint metrics, including only the material footprint (indicators 8.4.1 and 12.2.1). Few studies undertake city footprint assessments in the context of reporting on the SDGs. While poorly represented in the official indicator framework, clear linkages are apparent between a range of footprint metrics and the goals, targets and indicators of the SDGs. Vanham et al.^15^ highlight that a range of SDG targets and indicators could be monitored though environmental footprints, including fine particulate matter, chemicals, nitrogen, phosphorus, grey water, blue water, green water, land, carbon, biodiversity, and ecological. In Fig. 3, we expand on this analysis, highlighting linkages between specific goals, targets and indicators from the SDGs and corresponding footprint metrics. This identifies 14 specific footprint indicators that have the potential to support monitoring and assessment for 10 goals, 23 targets and 26 indicators from the SDG framework. This covers around 14% of official SDG targets where city footprint studies could contribute to monitoring the SDGs.

The fourth ring in Fig. 3 (City Footprint Literature) provides the number of footprint studies published that may be of relevance for assessing progress on different SDG targets and indicators. Studies relating to carbon (1488), ecological (903), and water (372) footprints are most advanced (highlighted in green), as well as energy footprint studies (86). Far fewer city-level studies were found relating to other footprint indicators, such as materials, land, chemicals, nitrogen/phosphorus, particulate matter, biodiversity or social footprints. While these numbers are indicative, this analysis highlights the great potential for city footprint studies to assist with assessing progress on the SDGs, as well as the relative maturity of research in different city footprint indicators.

Since footprint indicators are consumption-based metrics, they naturally provide useful information for SDG 12 which aims at ensuring sustainable consumption (and production) patterns. SDG target 12.8 generally highlights the need for citizens to have access to information and indicators that help to understand the complexity and urgency of the sustainable development challenge, something that footprints have been designed from the beginning to provide^54–56^. The wider consequences of city residents’ lifestyle choices are best described with footprint indicators, as demonstrated by many CF studies^8,9,64^. Shittu^72^, p.1 points out that urban households face specific challenges in the context of SDG 12 through ‘*[…] intensifying household consumption; rising commodification of household activities; continued reliance on unsustainable energy sources; low levels of sustainability education; high costs of sustainable lifestyles; and class differences in sustainable consumption patterns*’.

Assessing the water footprint of cities can also provide crucial information for SDG 6, in particular on water-use efficiency and water scarcity (SDG 6.4) and integrated water resources management and transboundary cooperation (SDG 6.5). Hoekstra et al.^73^ note that SDG 6 does not include any target on using green water more efficiently. WF assessments may also help expanding international cooperation between developed countries that consume high volumes of virtual water and developing countries where this water is extracted and where capacity for water efficiency, recycling and reuse can be improved (SDG 6.a)^73^. Through the use of lifecycle assessment of targets relating to SDG 6 on water and sanitation, Sørup et al.^52^ conclude that the SDGs are possibly conflicting with PBs.

### Benchmarking footprints against PBs

Footprints become more policy relevant if they are able to indicate absolute sustainability, i.e. whether or not consumption is sustainable^74^. This can be achieved by benchmarking them against sustainability thresholds as suggested by the safe and just operating space (SJS) framework^19^, also called the concept of doughnut economics^18,75^. Several conceptual approaches that link consumption-based accounts (CBA) to PBs have been described for environmental footprints^15,17,46^. The final outer ring in Fig. 3 shows that six of the nine PBs correspond to the SDGs (excluding stratospheric ozone, ocean acidification and novel entities). While a comprehensive list of footprint indicators was already mapped and benchmarked against PBs in 2015^74^, few studies explore the linkages between the SDGs, PBs and footprints^20^. Vanham et al.^15^ show that all planetary boundaries control variables can be assessed by an integrated family of environmental footprint indicators and how they can also be used to measure progress on the SDGs. However, for some footprints, e.g. water, locally relevant thresholds are equally relevant to planetary boundaries^15^.

Benchmarking at the national or any other spatial scale requires an approach to downscale global PBs^16,46^. This entails allocating a share of global safe operating space to sub-global entities—cities, regions, countries, companies or individuals—and may be based on socio-economic, ethical or political considerations, involving normative choices, or on bio-geo-physical principles^46,76^. Examples for normative criteria used in top-down approaches include equality (equal share per capita), needs (e.g. nutrition^77^), right to development (e.g. poverty or development status), sovereignty (e.g. land area or economic throughput), capability (e.g. income or resource efficiency) or responsibility (historical or current share of resource use, grandfathering)^78^, sometimes in different variations and combinations^76,79^. Bottom-up approaches to deriving local limits of resource use are based on specific environmental conditions of the locality, e.g. on local water availability or pollution, and may not be linked to global PBs^46,80,81^. At the national level, these concepts of benchmarking environmental footprints against PBs have been empirically demonstrated for many countries and regions^19,78,82^, even though there is currently no harmonised approach of allocating locally safe operating spaces^46^. Generally, these studies find that, from a consumption perspective, no country operates completely within a global safe operating space and that climate change and biogeochemical flows are the most transgressed boundaries.

Consumption-based footprint accounting can also be used to allocate the exceedance of a particular planetary boundary in a particular location to the consumption of goods and services in cities or other regions, e.g. for phosphorus use in agriculture^83^ or for excessive water extraction (measured against local water thresholds)^80^. This means that only the unsustainable share of an activity is accounted for, resulting in exceedance footprints, which indicate unsustainability as soon as they are larger than zero, requiring less interpretation. Currently, however, there is still a considerable degree of uncertainty and data scarcity related to localised exceedance of PBs.

### Footprints and PBs in absolute city-scale SDG assessments

At the city level, only very few studies have conceptualised or empirically implemented an assessment of SDG-relevant indicators by using consumption-based accounting benchmarked against PBs. An early study combines socio-economic targets derived from Millennium and Sustainable Development Goals (MDGs and SDGs) with bio-physical boundaries derived from PBs^47^. Progress metrics for five global cities include EF and local and embodied water, land and nitrogen use as well as emissions. This provides a means to view planetary and local boundaries as development targets from a metropolitan perspective. However, the definition of footprint scope in this study remains unclear.

Kawakubo et al.^3^ develop and apply a framework for assessing the sustainability of 76 cities across 39 countries, using SDG indicators to evaluate quality attributes combined with the absolute amount of GHG emissions per capita permissible under climate targets. This provides an assessment of the consumption levels commensurate with a reasonable quality of life to achieve the SDGs and with limiting global warming to 1.5 or 2.0 °C above pre-industrial levels. Fang et al.^67^ introduce natural capital stocks for land and water as locally relevant environmental boundaries for the Chinese city of Guiyang. The usually flow-based EF and WF indicators are thus extended with a stock-component that quantifies the length of time required to replenish the natural capital consumed in one year. The results confirm the city’s unsustainability on both accounts.

The EF was designed from the beginning to be benchmarked against the regenerative availability of natural resources, measured as biologically productive area^84,85^. Many city EF studies since 1996 have presented per-capita EF results, which can be benchmarked against global per-capita biocapacity^57^. Several recent papers compare the EF of cities in Portugal^86^ and Poland^57^ to the local biocapacity or environmental carrying capacity of their own, territorial endowment of natural resources. Protecting and restoring local, urban ecosystems and biodiversity directly contributes to SDG targets 11.4 and 11.7 and SDG 15. The results confirm considerable overshoots as cities rely on large amounts of hinterland resources. This assessment is arguably of limited value, since applying the same analysis to a rural area with large biocapacity would likely show that residents there live sustainably as they do not use up all locally available resources, even if their per-capita EF is the same or larger than that of city residents. This demonstrates that a scale for localised planetary boundaries needs to be chosen that makes absolute sustainability assessment meaningful and comparable.

Related to SDG 6 (sustainable management of water), a recent study calculates the EF and WF of urban water supply systems of seven cities around the globe^87^. Benchmarking the results against global biocapacity and the global water planetary boundaries shows that the systems of six cities exceed the global per-capita carrying capacity for ecosystems and water.

The Creating City Portraits approach^75^ suggests to view and assess a city’s performance under its own distinctive circumstances through four lenses that span across the social, ecological, local and global dimensions. Assessing global ecological responsibility in this approach entails downscaling of global PB data to the city scale based on equal per-capita shares and downscaling of national environmental footprints per capita, adjusted by average city household income. The concept was trialled in Philadelphia, Portland, and Amsterdam, with semi-quantitative results available for Amsterdam^75^. The first study that calculates seven different footprint indicators corresponding to five biophysical boundaries for a large number of cities was presented in 2020 by Hachaichi and Baouni^88^. Taking advantage of the efficiency of environmentally extended, multi-region input-output analysis^14^, the study presents per-capita footprints of 62 Arabian cities of the Middle East and North Africa region and compares them to national-level per-capita footprints. However, the study does not benchmark the footprints against PBs, nor does it relate them to specific SDGs.

From eight papers matching search terms on city social footprints, none explicitly calculates social performance from a CBA perspective^47,75^. Nor has a concept of benchmarking social footprints against SJS thresholds yet been proposed. O’Neill et al.^19^ measure social performance of countries against doughnut benchmarks, however, they use territorial rather than consumption-based indicators. While issues such as youth employment, health and other services, or security are clearly more locally relevant for city residents, several social footprint studies show that the principle of global responsibility can and should also be applied to topics such as wage income, forced labour, occupational health and safety or corruption^89–92^. Xiao et al.^93^ explicitly demonstrate how CBA of social impacts relates to SDGs for the examples of gender equality (SDGs 5, 8.5, 8.8), mother and child health (SDGs 3.1, 3.2) governance (SDGs 16.5, 16.6) and access to clean water (SDGs 6.1, 6.2). These metrics inform city stakeholders about the outsourcing of social problems to other places in the world.

## Research directions and policy implications

Neither the consumption perspective nor scientific assessments related to planetary boundaries play any significant role in existing city SDG measurement frameworks. There is considerable scope for footprint metrics to be used for more effective monitoring and benchmarking of city performance with respect to national and global targets and to help design accurate and successful local policies and interventions commensurate with the SDGs. The city footprint literature confirms the extensive reach and scope of footprint indicators to support monitoring of progress on the SDGs at city or other scales. Emerging studies that benchmark footprints against planetary limits facilitate greater understanding of the absolute sustainability of this progress. Thus, CBA enables cities to exploit strategies for both efficiency and sufficiency, providing a considerable opportunity to influence and set targets on consumption patterns rather than just comparing their relative progress or performance^35^.

### Implications for policy and practice

City-level sustainability assessments should enable local government and administrators to understand critical aspects of a city’s social, economic and environmental performance so that cities can be planned and managed to provide for livelihood and equity concerns of all inhabitants while ensuring that environmental pressures do not exceed key thresholds. The SDGs can support such decisions by providing a broadly legitimate, goal-oriented framework and dashboard of targets and indicators that have better coverage of social inequality aspects than previous frameworks^37^. As emerging studies highlight, there is also great potential for the inclusion of footprint metrics in city-scale SDG assessments to address the responsibilities of different city stakeholders, including city authorities as well as consumers^35^.

Given that cities are the main drivers of global consumption of goods and services, the noticeable failure to consider consumption-based impacts of cities or coherence with environmental thresholds represents an important missed opportunity for influencing sustainable urban development. This review shows that current sustainability assessment frameworks and indices for cities do not sufficiently address the global-local integration. Footprint indicators are one way to re-establish this connection. Indicators included in current city SDG assessments correspond more to supply-side priorities for local planning such as the delivery of services, sustainable infrastructure and transport, equal opportunities for work and education, etc. However, in the context of achieving national and global sustainability objectives, it is also critical that cities consider their consumption impacts that extend far beyond their borders. Including footprint indicators in city-level SDG assessments opens untapped opportunities to consider demand-side measures for influencing behavioural change in urban lifestyles^64^ and to enable more meaningful target setting by measuring performance based upon absolute environmental thresholds and therefore to raise the level of ambition.

By accounting for and raising awareness of impacts embodied in consumption^94^, footprint metrics open the door for new policies addressing urban consumption, city supply chains and procurement. In the literature, different forms of carbon pricing policies are the most widely mentioned policy interventions, and inter-city carbon trading schemes have also been suggested^64,72^. Many other policies have been proposed, aimed at decarbonising energy systems and infrastructure, engaging various stakeholders, influencing consumption behaviour or supporting sustainability education^72^. However, given that climate change is urgent and the time frame to implement the SDGs is short, Shittu^72^, suggests that more practice- and outcome-oriented policies to achieve SDG 12 are needed, e.g. by directly involving households in intervention programmes.

While it has been recognised for some time that city Scope 3 emissions are significant^12^ and methods have advanced for assessing these, the adoption of CBA in practice has been lagging behind^64^. We see several reasons for this. First, it has taken until very recently for the scientific community to exactly define and agree on the various scopes of GHG emissions that can be attributed to cities, and the accompanying terminology^8,9^. Second, calculating city carbon footprints in a reliable and reproduceable way presents methodological challenges, as CBA relies upon on a broad range of economic and physical data, methodologically sound models, and specialist expertise which have associated cost and resource requirements. Third, there may be a reluctance from city stakeholders to employ CBA more widely as reducing city footprints will require substantial curbing or altering of the consumption of goods and services by city residents which may run contrary to the prevailing paradigm of economic growth^44^.

Greater uptake of footprint indicators linked to absolute target thresholds in city SDG assessments will require methodological advancements, collaboration and partnerships. Initiatives such as C40 Cities have demonstrated that cities can contribute to global sustainability objectives, providing analysis, data, best practices and approaches for achieving net zero emissions targets in line with global thresholds. Such an approach could be extended through the standardisation of accounting protocols for cities for other footprint indicators along with methods for downscaling PBs to benchmark city performance. It is very important, however, that a range of accounting approaches and metrics is used in any comprehensive city sustainability assessment, so that all major aspects of sustainability are covered. Focusing on one indicator or perspective alone may limit policy options, potentially leading to unintended consequences^95^.

Another challenge relates to the methods used for assessing progress and combining SDG indicators with footprint metrics in a way that is comparable, intuitive and easy to communicate. A range of approaches have been used in recent assessments, generally assigning equal weight to all indicators^22,25^ and ranking of city performance on a normalised scale. In the context of absolute sustainability, this is problematic, as overall performance may be assessed as ‘good’ or ‘sustainable’ despite consumption levels that considerably transgress planetary boundaries. The approach by Kawakubo et al.^3^ offers an important advancement by dividing a city’s performance score on a broad range of SDG indicators by a single carbon footprint metric. This approach could be expanded to incorporate additional footprint indicators into the measurement of environmental load to provide a more comprehensive assessment of a city’s performance on both the SDGs and PBs, effectively penalising SDG performance where cities transgress PBs.

Footprint indicators on their own are clearly not sufficient to evaluate the full synergies and trade-offs with all SDGs and PBs. Their unique consumption perspective provides all the benefits described above, but they do not represent local, territorial or production perspectives which provide other important insights (which are provided by many SDG indicators). Nor do footprint indicators cover the full scope of all SDGs and PBs as Fig. 3 clearly shows. In that sense, we argue that footprints are necessary but not sufficient metrics for city sustainability.

### A research agenda for city footprints and SDGs

There is a clear need for interdisciplinary research to assess, understand and help to mitigate impacts from urban consumption. This first requires continued collaboration between Earth system, natural, environmental, system and economic scientists to further shed light on the interconnections between urban activities, footprints and planetary boundaries, and to understand dynamic relationships and system responses^15,74^. This includes research on cities’ influence on global flows of production and consumption, the ensuing impacts on the local to global scale and how this impacts the SDGs^40^. Continued research on downscaling of PBs based on either normative or scientific criteria will help to evolve footprints into indicators of absolute sustainability that are more accurate, relevant and representative of urban impacts and capable of better pinpointing actual environmental damage, e.g. for eutrophication^80,83^ or water use^66^. Even though large ranges of uncertainty remain within the PB framework^16^, its usefulness for informing and guard-railing future sustainable pathways is undeniable^96^ and should be harnessed for urban development.

With the focus of GHG mitigation in cities having been on urban form and emissions accounting, more social research is needed to understand underlying drivers of individual and household consumption behaviour and their specific contexts and structures in cities^72^. Key social mechanisms and practices inform and lead to social and technical innovation and vice versa. Knowledge about these social dynamics is important to identify and enable possible interventions by local governments that aim at mitigating incentives for over-consumption, removing barriers to behavioural changes and designing and enabling transitions to sustainable lifestyles^97^. Footprint metrics, including on social issues as outlined above, can help to assess city performance from a global and holistic systems perspective. Research on social footprint indicators in the context of city sustainability should therefore be intensified. Complementing this, the SDGs provide a suitable framework to evaluate demand-side mitigation through the lens of human well-being^98^.

Further methodological improvements are required to increase the accuracy of city-scale footprint calculations. Most studies rely on national-level data or models and employ various approaches for downscaling to the city level, which imposes limitations and increases uncertainty^9,99^. Deriving city-scale input-output tables and associated environmental and social satellite accounts is a relatively new, but yet to be improved, field of research that promises to better capture urban idiosyncrasies and supply chains of regional to global reach^99^. At the same time, more research on refining and harmonising methods for downscaling and assessing planetary boundaries to locally relevant levels is required.

The limited coverage of SDG-CBA-SJS interlinkages in the literature, especially at the city level, suggests that this remains very much an emerging field of research with great potential to capitalise on the legitimacy and integrated scope of the SDG framework underpinned by scientific methods for more comprehensive evaluation of progress. Cities will always be dependent on the world around them and therefore cannot be sustainable on their own. But they are essential vanguards in driving global sustainability towards a safe and just operating space^17,75^.

## Method

### Scope of the review and selection of references

This Review has focused on the literature relating to cities, footprints, SDGs and PBs. To identify relevant literature, a review protocol was used based on a set of query terms and conditions, starting broadly across the literature covering all of these topics, and then narrowing down to specific thematic areas and footprints. The review was conducted by searching the Web of Science (WoS) database, which was selected because it includes 24,000+ journals across 254 subject disciplines and is curated by expert in-house editors to include only journals that demonstrate high levels of editorial rigour and best practice. Details of the search terms and settings, as well as quantitative results shown in Figs. 1–3 are provided in the Supplementary Information.

The analysis of the literature focused on the most recent articles published since 2015, which coincides with the adoption of the SDGs. In addition to the scientific literature, some key references from the grey literature were also sourced, particularly relating to the cities SDG index published for European cities and the USA^4,5^.

### Exclusions and limitations

The query protocol adopted is broad and not all articles returned by WoS would be of specific relevance to this study. The numbers are indicative only and given the large volume of articles on footprints, not all of them were scanned for relevance. The literature review and query protocol did not include literature on urban metabolism which typically accounts for physical flows related to cities. This can provide important information about overall urban-natural system interactions^100^ and may include the transboundary use of materials and energy. However, this is different from the unique consumption perspective of the footprint metric, which also quantifies virtual flows and impacts outside a city, related to its consumption.

## Supplementary information

Description of Additional Supplementary Files

Supplementary Data 1
