# Supplementary material for: City footprints and SDGs provide untapped potential for assessing city sustainability
Source: Nat Commun. 2021 Jun 18;12:3758. doi: 10.1038/s41467-021-23968-2 (PMC8213854; doi:10.1038/s41467-021-23968-2)
Supplement: Supplementary file 1 — Description of Additional Supplementary Files [file 41467_2021_23968_MOESM1_ESM.pdf]

Description of Additional Supplementary Files

File Name: Supplementary Data 1
Description: This Supplementary Data contains the specific search strings that were used on the Web of Science to generate data used in the figures and text of the article.
